# Content Validation of the Prenatal Evaluation and Referral for Lactation (PEARL) Tool Using a Delphi Method

**DOI:** 10.3390/healthcare14172831

**Published:** 2026-09-03

**Authors:** Mirine Richey

**Affiliations:** Center for Prevention & Early Intervention Policy, Florida State University, 1339 E Lafayette Street, Tallahassee, FL 32301, USA; m.richey@fsu.edu

**Keywords:** lactation, delayed lactogenesis II, lactation risk factors, milk supply, prenatal screening, secretory activation, tool validation, Delphi method

## Abstract

Background: Delayed onset of lactogenesis II or delayed secretory activation, defined as milk onset > 72 h postpartum, is a recognized barrier to successful breastfeeding initiation and duration. Prenatal identification and screening for risk factors associated with delayed secretory activation remains underutilized in maternal–child health services. This project aimed to develop and validate content for the Prenatal Evaluation and Referral for Lactation (PEARL) tool, a prenatal screening and referral instrument designed to identify lactation risk factors and guide anticipatory counseling and early referral to an International Board Certified Lactation Consultant (IBCLC) or breastfeeding medicine physician. Methods: Tool development was informed by a structured literature review conducted on 2 October 2024, across four databases (CINAHL, PubMed, Scopus, and Embase), identifying clinical and other health factors associated with delayed lactogenesis II. A three-round Delphi process was used to establish content validity through multidisciplinary expert consensus. Item-level content validity (ICV) was evaluated using a predefined consensus threshold (≥0.80), while average-measures Intraclass Correlation Coefficients (ICC) were calculated as a complementary assessment of interrater consistency. Following tool refinement, a pilot implementation training was conducted to obtain preliminary feedback regarding clarity, usability, and implementation. Results: Twelve lactation experts were selected from 59 eligible respondents to participate in the Delphi process. Most of the proposed items achieved the predefined content validity threshold (ICV ≥ 0.80) across the first two Delphi rounds, resulting in refinement and consensus on the final PEARL instrument. Interrater reliability improved from moderate agreement in Round 1 (ICC = 0.769; 95% CI 0.425–0.953) to excellent agreement following refinement in Round 2 (ICC = 0.911; 95% CI 0.787–0.979). Qualitative feedback informed revisions to item wording, organization, and content. A pilot implementation training indicated that the tool was perceived as understandable, usable, and appropriate for prenatal practice. Conclusions: The PEARL demonstrated strong evidence of content validity and preliminary implementation feasibility as a standardized prenatal lactation screening tool. Future research should evaluate additional psychometric properties, implementation outcomes, and prospective clinical performance to determine its effectiveness in improving referral practices and breastfeeding outcomes.

## 1. Background

Breastfeeding is recommended to begin within 30–60 min after birth, to continue exclusively for the first six months, and then continued until the second birthday or after [1,2]. Breastfeeding is a public health priority both in the United States [3] and worldwide [4]. U.S. Healthy People 2030 breastfeeding goals include increasing exclusive breastfeeding rates (no infant formula or other complementary foods) from 25.6% to 42.4% at six months of age and breastfeeding duration goals to increase from 35.3% to 54.1% of infants receiving breastmilk until 12 months in addition to complementary foods [3]. Additional recommendations include the encouraging of breastfeeding duration for two years or beyond, as was seen in a policy update from the American Academy of Pediatrics, and includes critical recommendations that influence success, such as healthcare support [1].

Human milk feeding may be directly from the breast (breastfeeding) or by mechanical expression (pumping), both of which are of the same physiological process involving milk production referred to as lactation.

Approximately 60% of women stop lactating earlier than planned for many reasons, including health and medical complications (primary factors), which can arise early, during the onset of milk production referred to as secretory activation or lactogenesis stage II (LII) [5]. LII success depends on optimal levels of the lactation hormone prolactin, which is produced in response to the drop in progesterone levels when birth has been completed with the expulsion of the placenta during the third stage of birth. While many factors can disrupt breastfeeding and lactation duration, some complications can interrupt the LII process early on, which is considered the first 72 h after birth, affecting milk supply [6]. Milk supply is reported to be the biggest concern among new parents for continuing breastfeeding [7,8]. Several modifiable and non-modifiable factors, including medical conditions, hormonal imbalances, and maternal breast anatomy, can influence lactation success and are identifiable during pregnancy; however, despite their importance, systematic methods of prenatal screening for these risk factors remain underutilized, with other validated prenatal tools focusing on self-efficacy and self-assessment [9,10].

Lactation care therapeutics are extremely lacking in development and availability, and although potentially promising medication is in development phases that could quickly resolve milk supply issues [11], for now most women with early milk supply concerns, prevention and early anticipatory guidance by expert lactation professionals such as breastfeeding medicine clinicians or international board certified lactation consultants (IBCLC) are the most available strategies [5].

The 2022 American Academy of Pediatrics (AAP) breastfeeding recommendation highlights the need for a proactive approach to lactation care and increasing tools to improve outcomes that health and medical professionals can use, and reduce lactation complications [1]. Prenatal care can be an optimal time for assessing and exploring feeding care plans and assessing for potential lactation complications. Many health organizations, such as the Association of Obstetricians and Gynecologists (ACOG) recommend breastfeeding education and assessment as a part of routine prenatal care [12,13].

The Academy of Breastfeeding Medicine (ABM) recommends that hat breastfeeding support begin during prenatal care and that routine prenatal assessment include evaluation of maternal health conditions and breast anatomy that may affect lactation [14]. A prenatal breast examination should identify surgical scars (consistent with breast augmentation, breast reduction, or any other anatomically altering surgeries or trauma), as well as assessing breast asymmetry for any suggestions of breast hypoplasia (insufficient glandular tissue), as these conditions are known to impact milk supply, specifically during LII. Both ACOG and ABM recommend that women with these risk factors receive early referral to or ongoing supervision by a skilled lactation care provider to monitor milk production and address potential breastfeeding challenges. In addition to structural breast abnormalities, metabolic and hormonal conditions are also recognized contributors to delayed or impaired lactogenesis II.

Previous prenatal breastfeeding screening efforts, notably from earlier researchers such as Kathleen (Kay) Hoover and Dr. Verity Livingstone, focused on identifying factors that influence breastfeeding initiation, continuation, and success to be used by providers. Their work contributed to a foundational understanding of the importance for the need of developing screening tools that assess maternal intentions, knowledge, and potential barriers [15,16]. These screening efforts are highlighted as anchor references for the PEARL tool.

Establishing a theoretical framework for a new intervention in healthcare benefits from the use of established theories or a blending of theories to guide research to practice. The methodology for the PEARL content validation centers around a Delphi method [17,18], using Donabedian’s theory as appropriate framework [19]. In a systematic review proposing a new quality framework for assessment tools, Whiting et al. (2017) suggest a three-step process (initial steps, tool development, and dissemination) which aligns with the conceptual framework [19,20].

The Delphi method is commonly utilized in nursing and health research [17,20,21,22], particularly in identifying clinical priorities. Limitations of using the Delphi method, particularly for a health screening tool development process, include the need to provide future researchers and the user additional quality measures for critically evaluating the method and outcomes of the process [20,23]. This project aimed to develop and validate the content for the Prenatal Evaluation and Referral for Lactation (PEARL) tool by systematically gathering and synthesizing expert opinion through multiple Delphi rounds. This process supports the evidence-based development of a prenatal lactation risk screening and referral tool designed for use by prenatal providers and maternal–child health professionals.

## 2. Methods

### 2.1. Study Aim, Design, and Setting

**Figure 1:**
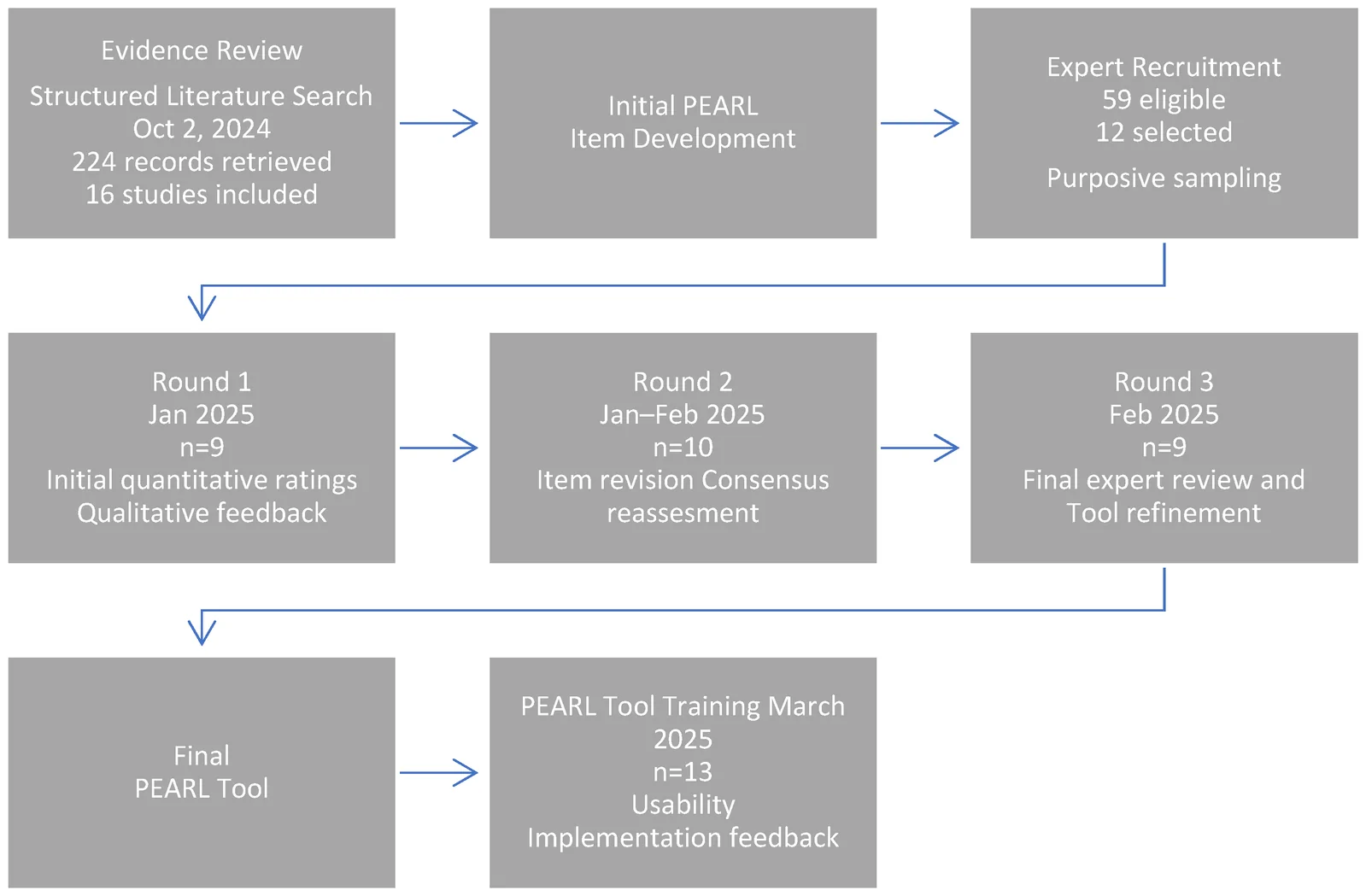
Development and Content Validation Process for the PEARL Tool.

The aim of this study was to develop and establish the content validity for the Prenatal Evaluation and Referral for Lactation (PEARL) tool, to be used for prenatal screening and referral to lactation care. The full timeline of the PEARL content validation process can be seen in Figure 1. A three-round Delphi design was used to achieve expert consensus on the relevance, clarity, and clinical utility of proposed tool content. The Delphi method is a structured, iterative process that anonymously collects and refines expert opinion over successive rounds to achieve convergence toward consensus [20,21,22]. Three rounds were planned a priori, as this is generally considered sufficient to refine proposed items, incorporate qualitative feedback, achieve stable consensus, and minimize participant burden and attrition during instrument development [17,21,22,23,24,25]. The first round evaluated the initial item pool and solicited qualitative feedback, the second round reassessed revised and newly proposed items, and the third round provided final review of the refined instrument.

A purposive sampling approach was used to recruit experts with diverse professional backgrounds relevant to prenatal lactation care. Twelve experts were selected because Delphi methodology emphasizes the breadth and quality of expertise rather than statistical sample size, with panels of approximately 10–18 participants commonly recommended for relatively homogeneous expert groups [21,22]. Recruitment continued until a multidisciplinary panel representing diverse clinical disciplines, practice settings, and lactation expertise had been assembled. The study was conducted entirely virtually using Qualtrics software (Qualtrics, Provo, UT., 2025), deployed through a institutional license from Florida State University, between January–February 2025.

The process adhered to the key Delphi principles of anonymity, iteration, controlled feedback, and a predefined consensus threshold of ≥80% established a priori. This approach was guided by Donabedian’s framework which emphasizes the structure, process, and outcome relationships in healthcare interventions [19] and aligns with recommended frameworks for tool development and dissemination in health research [18].

### 2.2. Participants and Recruitment

Panelists were recruited using purposive sampling to identify health professionals with substantial clinical expertise in lactation and maternal–child health. Interested respondents completed a screening questionnaire used to determine eligibility and characterize the expert panel. Information collected included years of lactation experience, academic training, professional licensure or discipline (e.g., physician, nurse, dietitian), lactation credentials (e.g., IBCLC, or Women, Infant and Children’s (WIC) peer counselor), areas of maternal–child health experience (pregnancy, labor and birth, postpartum), and primary practice setting (e.g., hospital, community-based programs, WIC. Participants also reported their practice location (United States or international), race and ethnicity, and the racial and ethnic populations they most frequently served. These data were collected to assemble a multidisciplinary panel with diverse clinical expertise and practice perspectives.

### 2.3. Evidence Review and Initial Item Development

The initial PEARL item pool was informed by a structured literature review conducted on 2 October 2024, to identify maternal health, pregnancy-related, hormonal, metabolic, and breast anatomical factors associated with delayed lactogenesis II that could be identified during the prenatal period. PubMed, CINAHL, Scopus, and Embase were searched without publication date restrictions using combinations of terms related to delayed lactogenesis II, or milk supply, breastfeeding or lactation, pregnancy or prenatal care, and relevant metabolic, hormonal, and breast anatomical conditions. Searches were limited to English-language studies involving humans. The search identified 224 records, which were imported into Covidence software for systematic reviews, provided by the Florida State University Maguire Medical Library, for deduplication and screening. After removal of 117 duplicates, 107 titles and abstracts were screened; 38 articles underwent full-text review, and 16 studies met the criteria for inclusion. Studies were included when they addressed risk factors identifiable during pregnancy with potential relevance to delayed lactogenesis II in the early postpartum period. Findings from the review, together with existing prenatal lactation assessment approaches and clinical guidance, (ACOG, AAP, ABM) were used to inform the initial domains and draft items presented to the Delphi panel.

### 2.4. Delphi Process and Data Collection

The study consisted of three iterative Delphi survey rounds that combined quantitative ratings with qualitative open-ended feedback. Surveys were administered and managed using the Qualtrics platform (Florida State University). The Delphi process incorporated the core principles of anonymity, iteration, and controlled feedback, with revisions between rounds informed by both quantitative ratings and qualitative comments, consistent with established Delphi methodology [24,26].

Following each survey round, quantitative ratings were summarized using descriptive statistics and item-level consensus was evaluated against the predefined content validity threshold. Qualitative comments were reviewed to identify recommendations for clarifying, modifying, adding, or removing items before the subsequent round. This iterative process allowed the PEARL tool to be progressively refined while maintaining transparency and consistency throughout the consensus process.

Expert selection followed the criteria described by Skulmoski et al., emphasizing participants’ knowledge of the topic, relevant experience, willingness to participate, and availability to complete multiple Delphi rounds [22]. The multidisciplinary composition of the panel, including variation in professional discipline, practice setting, academic preparation, and populations served, was intended to capture diverse clinical perspectives relevant to prenatal lactation care.

### 2.5. Statistical Analysis

Quantitative data were analyzed using SPSS version 29. Panelists rated each proposed item using a 3-point Likert scale (1 = Not important, 2 = Important, 3 = Very important). The 3-point scale was selected to encourage clear endorsement or rejection of proposed items while minimizing midpoint ambiguity. In a methodological evaluation of Delphi rating scales, De Meyer et al. concluded that a 3-point scale may be preferable when determining final consensus because additional response categories may increase response variability [17]. Previous Delphi studies have successfully used rating scales ranging from three to nine response options depending on the objectives of the study [27,28,29,30,31]. Because consensus thresholds and rating scales vary across Delphi studies, it is important to explicitly define the consensus criterion, response scale, and analytic approach to ensure methodological transparency [23,27]. Consistent with established content validation methods, ratings of 2 (Important) and 3 (Very important) were classified as agreement for calculation of the item-level content validity index (ICV), whereas ratings of 1 (Not important) were classified as disagreement [28,29,32]. The ICV was calculated as the proportion of raters who assigned a score of 2 or 3 to a given item divided by the total number of raters. Items achieving an ICV ≥ 0.80 were retained for subsequent Delphi rounds, whereas items below this threshold were removed or revised based on expert feedback [28,32].

### 2.6. Content Validity

The threshold for ICV was established a priori at ≥0.80 agreement per item, consistent with recommended consensus thresholds used in healthcare instrument development, including breastfeeding and infant feeding research [29,30].

### 2.7. Interrater Reliability Analysis

To complement the item-level content validity analysis, overall interrater reliability was assessed using the average-measures Intraclass Correlation Coefficient (ICC), which evaluates the overall consistency of ratings among multiple expert raters [33]. Although expert ratings were collected using a 3-point ordinal scale, the ICC was selected to assess consistency across the entire panel of raters rather than agreement for individual ordinal categories. In contrast, ICV provided item-level agreement based on dichotomized ratings. Together, these complementary analyses allowed evaluation of both overall interrater consistency and individual item agreement. Although alternative agreement statistics developed for ordinal data are available, ICC was selected because the objective was to evaluate the overall consistency of ratings among multiple expert raters, whereas ICV quantified item-level agreement for content validation. Interrater reliability was analyzed in SPSS version 29 using a two-way random-effects model with a consistency definition and average measures. A two-way random-effects model was selected because both the expert raters and the proposed instrument items were considered representative samples from larger populations, allowing the reliability estimates to be generalized beyond the study sample. Consistent with recommendations by Koo and Li, interrater reliability was interpreted using the ICC estimate, 95% confidence intervals, and the associated F statistic [33]. While ICV was calculated from dichotomized ratings (agreement/disagreement), the ICC analysis retained the original 3-point ratings to evaluate overall consistency among expert raters.

## 3. Results

### 3.1. PEARL Delphi Panel Summary

The recruitment survey received 59 eligible responses including 10 from the original purposive recruitment, and additional respondents recruited through professional lactation networks. Using the predefined selection criteria described in the Methods, 12 experts were purposively selected to assemble a multidisciplinary panel representing diverse clinical disciplines, practice settings, and lactation expertise while maintaining a panel size consistent with recommendations for Delphi studies. Selection prioritized years of lactation experience, educational background, certification, and representation across multiple clinical settings. The final panel included ten International Board Certified Lactation Consultants (IBCLCs), two physicians (family medicine and obstetrics), three registered nurses, one registered dietitian, one physical therapist, and one licensed mental health counselor (Table 1).

Five participants (42%) had published research in the field of lactation and 11 (91.7%) reported more than 10 years of professional lactation experience. The selected panel represented a broad range of practice settings, including hospital (inpatient and outpatient), home visiting, WIC, NICU, private practice, community-based or nonprofit programs, and volunteer peer-support organizations. Panelists reported experience working with a wide range of populations, including Black/Afro-Caribbean, Hispanic, Indigenous/Native, Asian, Pacific Islander, and White/Caucasian families. These characteristics supported the various clinical perspectives needed for the consensus process. Panelists were issued a study identification number for analysis purposes.

### 3.2. Round 1 Results

In Round 1, panelists evaluated the relevance of proposed prenatal to lactation screening items and provided qualitative feedback through open-ended response fields. Nine of the twelve raters participated in this round (Raters 1, 2, 3, 4, 6, 7, 10, 11, and 12).

Consensus was evaluated using the predefined ICV. Items achieving an ICV ≥ 0.80 were retained, whereas items below the threshold were removed or revised. Qualitative feedback was reviewed to clarify wording, refine existing items, and identify additional items consistent with the PEARL conceptual framework for inclusion in Round 2.

A high proportion of items (≥80%) reached consensus, indicating strong initial content validity. Table 2 presents the descriptive statistics generated in SPSS v29. The average-measures Intraclass Correlation Coefficient (ICC) was 0.769 (95% CI: 0.425–0.953), with a statistically significant F (6, 150) = 4.336, *p* < 0.001. While significant, this ICC value fell below the predefined 0.80 threshold, suggesting only moderate agreement and informing refinement.

Participation varied slightly, which is commonly reported in Delphi studies [34,35], (Round 1, *n* = 9; Round 2, *n* = 10; Round 3, *n* = 9.) Because Delphi methodology evaluates consensus at each round independently rather than requiring identical participants across all rounds, all responses from eligible panelists were included in the analyses for their respective rounds.

Agreement was defined as ratings of 2 (Important) or 3 (Very Important). Items with an item-level content validity index (ICV) ≥ 0.80 were retained for the subsequent Delphi round. Percent agreement was calculated using the number of panelists who rated each item. Items with lower agreement were revised or removed before Round 2 based on both quantitative ratings and qualitative feedback. This iterative refinement process strengthened consensus in subsequent rounds and represents a key feature of the Delphi methodology. Table 3 summarizes the ICV results from Round 1.

The corresponding F (6, 150) = 4.336, *p* < 0.001 was statistically significant; however, the ICC value of 0.769 (95% CI 0.425–0.953) indicates moderate interrater reliability but below the predefined 0.80 threshold for strong agreement, suggesting that additional refinement of the instrument was warranted before the subsequent Delphi round.

### 3.3. Round 2 Results

Instructions for Round 2 were similar to those in Round 1, with additional guidance reminding raters that the purpose of the PEARL tool is to screen during pregnancy specifically for potential risks associated with delayed secretory activation (lactogenesis II). For important concepts outside the scope of the tool, raters were encouraged to suggest other validated instruments that providers should reference (e.g., the Edinburgh Postnatal Depression Scale [EPDS] for depression screening [36]. Ten of the twelve raters responded to Round 2, seven of whom had participated in Round 1 (Raters 1, 2, 3, 4, 5, 6, 8, 9, 10, and 12).

The only categorical modification from Round 1 to Round 2 was the combining of the Breast Questions and Breast and Nipple Anomalies categories, as these domains demonstrated conceptual overlap.

Consensus was again evaluated using the predefined ICV threshold. Most items achieved an ICV ≥ 0.80, demonstrating strong agreement following refinement of the instrument. Qualitative feedback was used to further clarify item wording and finalize the content presented for the third Delphi round. Table 4 presents the interrater reliability statistics. The average-measures ICC for Round 2 increased to 0.911 (95% CI: 0.787–0.979), indicating excellent reliability. The corresponding F statistic, F (7, 112) = 11.284, *p* < 0.001, demonstrated that the observed agreement was improved compared with Round 1.

The ICC of 0.911 (95% CI 0.787–0.979) indicates excellent interrater reliability, F (7, 112) = 11.284 *p* < 0.001.

Table 5, below, summarizes the original categories, the questions that were generated in Round 1 and evaluated in Round 2. All items with achieving the predefined consensus threshold of ≥80% were kept and incorporated for the revised PEARL tool for the Round 3 final review.

### 3.4. Round 3 Results

The Round 3 survey was distributed after incorporation of revisions based on the quantitative ratings and qualitative feedback obtained during Rounds 1 and 2. Nine of the twelve panelists participated in the final round (Raters 1, 3, 4, 5, 6, 7, 10, 11, and 12). Unlike the first two rounds, Round 3 was designed as a qualitative confirmation review of the refined PEARL instrument rather than an additional quantitative rating round. Panelists were asked to review the final organization, wording, and clinical usability of the instrument and provide any remaining recommendations before finalization. No new items required consensus voting in Round 3; instead, the round consisted entirely of open-ended text boxes to collect final qualitative input. Several proposed items were consolidated, reworded, or relocated across conceptual domains during the iterative Delphi process. Consequently, the final number of items within each category does not necessarily equal the sum of the original and newly proposed items. The survey presented the final organization of categories and question order as they appear in the PEARL tool (Table 6), and the final layout of the PEARL tool is provided in the Supplementary Materials.

Qualitative responses from each survey round were thematically reviewed and organized within literature-informed domains: maternal health, pregnancy health, breastfeeding history, and breast anatomy. Comments suggesting potential new items were evaluated for conceptual relevance and alignment with the research question. Items meeting these criteria were incorporated into the subsequent Delphi round for expert review.

### 3.5. PEARL Tool Implementation Training

A finalized PEARL tool was introduced with a 1.5 h virtual training via Zoom (FSU) platform in March 2025. Continuing education credits in nursing and lactation were approved for the training through the FSU Center for Prevention & Early Intervention Policy training system (cpeip.fsu.edu). Thirteen participants attended the training and included those who expressed interest in the Delphi panel but were not recruited as panelists.

The training focused on implementation of the PEARL tool and was presented as a structured approach to assessing maternal medical history, pregnancy conditions, breastfeeding history, and breast and nipple anatomy. The training reviewed the evidence supporting each assessment domain and discussed referral pathways for individuals identified as having increased risk for delayed lactogenesis II.

Participants engaged in case-study scenarios using the PEARL tool, including structured history-taking to identify prenatal lactation risks. Case scenarios demonstrated the clinical utility of the tool in assessing patients with varying risk profiles, including those with lactation insufficiency concerns, gestational diabetes, and psychosocial stressors. The training concluded with a focus on practical implementation strategies, including the integration of the PEARL tool into routine prenatal care, establishing referral networks for lactation support, and leveraging creative approaches to care such as telehealth for prenatal breastfeeding education.

Qualitative feedback suggested that the PEARL tool was seen as a valuable resource for client interactions, though one nurse participant, who also identified as an experienced lactation consultant, expressed that some sections of the training, such as lactation physiology, were more relevant for “novice providers”, while another participant, a perinatal nurse, commented, “There were some pertinent things I had never thought of before. Including how some medical complications (HTN, GDM, high BMI and PCOS) may impede milk production. I will be starting with asking all of my prenatal families about breast changes after 16 weeks of pregnancy and going through more in-depth screening from there”.

Another nurse participant commented that she had just completed a 52 h course in lactation and that in the training she “relearned some things that were not 100% covered”. The contrast of comments reflects the support of standardized prenatal lactation assessment across providers with varying levels of breastfeeding knowledge and experience. Evaluation of implementation outcomes, provider adoption, and clinical effectiveness will require future prospective studies.

## 4. Discussion

The development and content validation of the PEARL screening tool demonstrate a structured, evidence-based approach to establishing expert consensus on prenatal lactation risk assessment. Through a three-round Delphi process, the multidisciplinary expert panel achieved strong agreement regarding the relevance of the proposed screening items, supporting the content validity of the tool. Interrater reliability analysis complemented these findings by demonstrating improved consistency of expert ratings following iterative refinement of the instrument.

A pilot implementation training tested and evaluated the clarity, usability, and potential implementation of the tool in prenatal practice. The evaluation from the pilot training including the comments, reinforced both the clarity and feasibility of the PEARL tool for practice while addressing an important gap in prenatal care. Participants reported confidence in the tool’s relevance and use, and the training process helped clarify its application within different clinical roles. Importantly, this phase also suggested that the PEARL tool may facilitate anticipatory guidance and care in a consistent manner.

The final PEARL tool (PEARL Supplementary Materials) incorporated items from current recommendations, literature review, and expert feedback from those who participated in the Delphi process, and considerations from those who participated in the tool training.

### 4.1. Qualitative Feedback

Qualitative feedback from each round provided valuable insights and facilitated new considerations and refined the tool wording, organization and content.

Many panelist recommendations resulted in refinement of item wording, organization, or the addition of new screening questions. For example, feedback supported the inclusion of prenatal questions addressing previous breast cancer, infertility or menstrual history, sexual trauma, high-risk pregnancy, multiple gestation, planned cesarean birth, and contraindicated medications. These additions strengthened the clinical relevance of the tool while remaining consistent with the literature and the conceptual framework established during item development.

Several recommendations were intentionally not incorporated because they extended beyond the scope of the PEARL tool such as postpartum breastfeeding management, workplace support, grief, depression, latch assessment, and milk transfer. Although the suggestions were aligned with the current literature and reflected clinical relevance, exclusion helped ensure that the tool remained concise in scope and appropriate during standard prenatal care visits.

One important contribution from the qualitative review was expanding the pregnancy history domain to include prenatal risk factors associated with obstetrical complications. As many of these risk factors present during pregnancy such as hypertension, placenta previa or accreta, a planned c-section birth, and multiple gestational pregnancies and are known to complicate breastfeeding initiation and increase challenges [37,38,39].

One notable outcome of the qualitative feedback was addressing breastfeeding goals, knowledge, and beliefs. Although psychosocial factors were not part of the original conceptual framework and are addressed in other validated prenatal tools [9,10] multiple panelists identified this area as important for noting in addition prenatal counseling. The item subsequently achieved consensus during Round 2 and was retained in the final instrument, illustrating the value of combining literature-informed item development with iterative expert review.

While there were many psychosocial factors that raters commented on (workplace, supports, depression, grief), one panelist summarized the consensus scope as “In an attempt to keep this screening tool simple, you may choose to exclude areas that other health professionals are already addressing. Avoid duplication but remember the social situations, including lack of help and returning to work, are major factors to the ongoing duration of breastfeeding”.

Qualitative feedback also informed implementation of the PEARL tool. Several panelists requested clarification regarding the intended users of the instrument, leading to revisions that explicitly describe the tool as appropriate for physicians, nurses, home visitors, lactation professionals, health educators, and other prenatal care providers. These revisions, together with the accompanying implementation training, support the use of the PEARL as a provider-administered screening tool rather than a patient-completed questionnaire or self-assessment [12,13,14].

### 4.2. Final Tool

Tool features that met consensus included having the tool available in plain language versus medical terminology where possible. Medical, health, and anatomical terms are used, and facilitating implementation training would provide further details and clarification for novice users as a provider-facing screening tool. The final Prenatal Evaluation and Referral for Lactation (PEARL) tool is located in the Supplementary Materials.

Tool features such as format, length, and layout did not appear to be a primary concern among Delphi participants. While raters were asked to evaluate the importance of practical considerations, such as keeping the tool to one double-sided page and ensuring both printed and electronic versions would be available, they received slightly lower levels of agreement (78%) than other tool elements. This suggests that while these features were relevant, most panelists did not see them as essential. In contrast, specific structural features that supported clinical use were rated highly. For example, the inclusion of an item confirming screening for depression and psychosocial impacts, as well as a breast diagram for documenting surgical scars, anatomical anomalies, or other physical concerns, received strong endorsement (89–100%).

The final version of the PEARL tool is two printed pages long. Initially, the first version of the PEARL was a binary yes/no scoring system for the provider with the questions phrased in language friendly to the patient. With the consistent and numerous comments from the raters on issues that influence the entire course of lactation, a third option was added.

While the screening is essentially a binary yes/no in terms of risk factors for referral, the Breastfeeding Education column was added in the final version to incorporate the rater’s feedback. Whereas most of the items requested from the raters are addressed in breastfeeding education and support, this allows the provider to recognize that some of the items may not need an expert referral but also serves as a reminder that breastfeeding education should be part of usual care. The provider can include breastfeeding education if that is part of their role, or the patient can be referred to a structured support group or class.

### 4.3. Strengths, Limitations, and Future Research

The primary strength of this study is seen in the established content validity through a structured, three-round Delphi process involving a multidisciplinary panel of experts with extensive experience in lactation and maternal–child health. The iterative combination of quantitative consensus ratings and qualitative feedback allowed refinement of the PEARL tool while incorporating diverse clinical perspectives from hospital, community, public health, research, and private practice settings. In addition, the pilot implementation training provided preliminary evidence regarding the clarity, usability, and feasibility of introducing the tool into prenatal practice.

Several limitations should be considered. First, although the Delphi panel represented diverse clinical disciplines and practice settings, the number of participants was relatively small and varied across rounds, which is common in Delphi methodology where expertise rather than sample size is emphasized. Although content validity was achieved, this does not confirm the psychometric validation.

Finally, the implementation training was designed to evaluate educational implementation and gather preliminary user feedback rather than determine clinical effectiveness. Future research should focus on prospective implementation studies evaluating the PEARL tool in routine prenatal care across multiple healthcare settings.

## 5. Conclusions

The PEARL tool demonstrated strong content validity through a structured Delphi process, supported by high interrater agreement (ICC) and positive feedback from a brief pilot training with maternal and child health providers. Its concise format balances usability with clinical comprehensiveness, enabling efficient integration into a variety of prenatal care workflows.

While content validation is an essential first step, future research should include broader reliability testing and pilot implementation to assess usability, feasibility, and impact on breastfeeding outcomes.

The PEARL tool provides one of the first evidence-informed, consensus-based provider-based prenatal screening instruments specifically designed to identify women at increased risk for delayed lactogenesis II and facilitate timely lactation education and referral before the baby is born. As prenatal lactation support continues to receive greater emphasis within maternal–child health, standardized screening tools such as the PEARL may help strengthen early identification, improve coordination of care, and ultimately support breastfeeding outcomes across diverse populations.

## Figures and Tables

**Table 1:** Characteristics of Delphi Expert Panel (*N* = 12).

Characteristic	*n* (%)
Highest educational attainment	
Bachelor’s degree	2 (16.7)
Master’s degree	8 (66.7)
Doctoral degree	2 (16.7)
Professional discipline/credentials	
International Board Certified Lactation Consultants (current or retired)	10 (83.3)
Physicians (Family Medicine, Obstetrics)	2 (16.7)
Registered Nurses	3 (25.0)
Registered Dietitian	1 (8.3)
Physical Therapist	1 (8.3)
Licensed Mental Health Counselor	1 (8.3)
Years of experience >10	11 (91.7)

Note. Categories are not mutually exclusive; panelists represented multiple disciplines and credentials.

**Table 2:** Intraclass Correlation Coefficient Agreement of Round 1 (*N* = 9).

Measure	ICC	95% CI (Lower–Upper)	F (df_1_, df_2_)	*p* Value
Average measures	0.769	0.425–0.953	4.336 (6, 150)	<0.001

Note. ICC = Intraclass Correlation Coefficient; CI = confidence interval. Statistical significance was defined as *p* < 0.05. Consistent with the predefined study criteria, an ICC ≥ 0.80 was considered indicative of strong interrater reliability.

**Table 3:** Item-level content validity for Round 1 (*N* = 9).

Category	Question	Kept	Agreement *n*/*N* (%)
Breastfeeding History	Is this your first baby?	Yes	(9/9) 100
	Do you have other children?	Yes	(8/9) 89
	Did you breastfeed your other children?	Yes	(9/9) 100
	How did breastfeeding go for you?	Yes	(9/9) 100
	Follow-up question if negative experience reported: What was the resolution to the issues?	Yes	(9/9) 100
Maternal Health	Diabetes Type 1 or Type 2	Yes	(8/9) 89
	Polycystic Ovarian Syndrome (PCOS)	Yes	(9/9) 100
	Type of family planning method preferred or to be used after birth (hormonal, barrier, LARC, etc.)	Yes	(9/9) 100
	History of hormonal medication use (testosterone, progesterone, estrogen, other)	Yes	(9/9) 100
	History of thyroid disorder	Yes	(8/9) 89
	Pre-pregnancy BMI	Yes	(8/9) 89
	Maternal age	Yes	(9/9) 100
Pregnancy Health	Current BMI	No	(7/9) 78
	Gestational weight gain (low, normal, excessive)	Yes	(8/9) 89
	Gestational diabetes	Yes	(9/9) 100
	Hypertensive disorder	Yes	(9/9) 100
	Preterm risk factors	Yes	(9/9) 100
Breast Questions	Have you noticed breast changes during this pregnancy?	Yes	(9/9) 100
	Have your breasts/chest experienced any trauma, burns, or radiation?	Yes	(9/9) 100
	Have you had any breast/chest surgery?	Yes	(9/9) 100
	Place of incision(s): armpit, under areola, under breast, across breast, other	Yes	(9/9) 100
	Do you have normal nipple sensation/reactivity since the surgery?	Yes	(9/9) 100
	Breast hypoplasia (under-developed): Left, Right, Both	Yes	(9/9) 100
Breast/Nipple Anomaly	Extra nipples on or near nipple-areolar complex	No	(7/9) 78
	Extra breast tissue/accessory breast (location)	No	(6/8) 75
	Inverted nipples (that do not evert with compression)	Yes	(9/9) 100
**Tool Features**	An illustration of two breasts to note surgical scars, anomalies, concerns	Yes	(8/9) 89
	Language on how to make a referral for additional care	Yes	(9/9) 100
	No more than one page front/back (importance)	No	(7/9) 78
	Available as a printed tool (importance)	No	(7/9) 78
	Available electronically (importance)	No	(7/9) 78
	Available in plain language vs. medical terminology	Yes	(8/9) 89
	Confirming screening for depression	Yes	(9/9) 100

Note. Percent agreement was calculated using the number of panelists providing a rating for each item (*N*). Denominators vary slightly because some participants did not respond to every question.

**Table 4:** Intraclass Correlation Coefficient Agreement of Round 2 (*N* = 10).

Measure	ICC	95% CI (Lower–Upper)	F (df_1_, df_2_)	*p* Value
**Average measures**	0.911	0.787–0.979	11.284 (7, 112)	<0.001

Note. ICC = Intraclass Correlation Coefficient; CI = Confidence Interval.

**Table 5:** Round 2 Results for Additional Items (*n* = 10).

Category	Added Item from Qualitative Feedback	Kept	Agreement *n*/*N* (%)
Breastfeeding History	Add a question relating to breastfeeding goals/knowledge/beliefs	Yes	10/10 (100)
Maternal Health	Add a question about past breast cancer diagnosis	Yes	10/10 (100)
	Add a question about previous infertility/menstrual history	Yes	9/10 (90)
	Add a question relating to sexual abuse/trauma	Yes	10/10 (100)
Pregnancy Health	Experiencing high stress	Yes	8/10 (80)
	Pregnant with twins/multiples	Yes	9/10 (90)
	Known placental complications	Yes	8/10 (80)
	Planned Caesarean birth	Yes	9/10 (90)
	Birth defect diagnosis in baby (e.g., oral/facial abnormalities)	Yes	8/10 (80)
	Use of lactation-contraindicated medications	Yes	9/10 (90)
Breast Questions/Nipple Anomalies	Add a sub-question on breast symmetry	No	7/10 (70)
	Add a sub-question on surgery types (specify)	No	7/9 (78)
	Reduction mammoplasty with or without nipple removal	Yes	9/9 (100)
	Breast augmentation as a separate question under surgery type	Yes	9/9 (100)
	Clarify inverted-nipple question (everts with compression or graspability)	Yes	8/9 (89)
	Add a sub-question on bifurcated nipple	No	6/9 (67)
Tool Features	Add a box for referral or confirmation of breastfeeding education/support group	Yes	10/10 (100)

Note. Agreement was defined as ratings of 2 (Important) or 3 (Very Important). Items with an item-level content validity index (ICV) ≥ 0.80 were retained for Round 3. One panelist did not respond to several breast-related items; therefore, denominators vary across items.

**Table 6:** Round 3 Final PEARL Content Summary (*N* = 9).

Category	Round 1 Initial Items	Round 2 Items Added	Final PEARL Items
**Maternal Health History**	7	3	10
**Pregnancy Health** **History ^1^**	5	7	5
**Breastfeeding History**	5	1	6
**Breast Questions ^2^**	6	5	8
**Nipple Questions (merged on round 2 with Breast Questions)**	3	n/a	n/a
**Tool Features**	7	1	6

Note. ^1^-Although seven additional pregnancy-related items were proposed during Round 2, only those achieving the predefined consensus threshold (ICV ≥ 0.80) were retained in the final instrument. ^2^-During Round 2, the Breast Questions and Breast/Nipple Anomalies categories were merged into a single Breast Questions category.

## Data Availability

The original contributions presented in the study are included in the article/Supplementary Material. Further inquiries can be directed to the author.

## Supplementary Materials

The following supporting information can be downloaded at https://www.mdpi.com/article/10.3390/healthcare14172831/s1: Prenatal Evaluation and Referral for Lactation (PEARL).
