# Supplementary material for: Content Validation of the Prenatal Evaluation and Referral for Lactation (PEARL) Tool Using a Delphi Method
Source: Healthcare (Basel). 2026 Sep 3;14(17):2831. doi: 10.3390/healthcare14172831 (PMC13565660; doi:10.3390/healthcare14172831)
Supplement: Supplementary file 1 [file healthcare-14-02831-s001.zip › healthcare-4367233-supplementary.pdf]

Prenatal Evaluation and Referral for Lactation (PEARL)

Refer to an IBCLC lactation consultant or breastfeeding medicine provider if responses are in the **Risk Factor** column.<br><br>Refer for usual care and education for factors in the **Breastfeeding Education** column.		**Breastfeeding Education**	**Risk Factor for Referral**
**Maternal Health History**<br><br>*Note:* Maternal age (>35) and health history can influence lactation success. Additional factors to screen and refer for are below.			
Is there a history of breast cancer?	No		**Yes**
Is there a history of diabetes? (Type I Type II)	No		**Yes**
Was the pre-pregnancy BMI >30?	No		**Yes**
Were there documented hormonal disorders before pregnancy? (PCOS, infertility, menstrual disorders)	No		**Yes**
Is there a history of thyroid disorder?	No		**Yes**
Is there a history of hormonal medication use? (testosterone, progesterone, estrogen)	No		**Yes**
Is the preferred family planning method type:	Non-hormonal	Hormonal	
Has patient screened positive for depression, trauma or abuse?	No		**Yes**
Document any additional screening tools used if any: EPDS, PHQ-9, ACEs, other:			
**Pregnancy Health History**<br><br>*Note*: Some conditions detected during pregnancy may influence lactation success. Referral during pregnancy can establish anticipatory guidance from an IBCLC lactation consultant or breastfeeding medicine provider.			
Is the gestational weight gain for BMI:	Low or normal		**Excessive**
Is there a current diagnosis of gestational diabetes?	No		**Yes**
Is there a current diagnosis of hypertension or preeclampsia?	No		**Yes**
Are there known preterm birth risk factors? (high stress, multiples, birth defects, placenta complications, planned cesarean birth, other)	No		**Yes**
Is there current or potential use of medications contraindicated for lactation or milk production?	No		**Unsure/Yes**

Breastfeeding History

Note: Breastfeeding education is recommended starting in pregnancy and a referral to an IBCLC lactation consultant or breastfeeding medicine provider is recommended for previous or anticipated lactation complications.

Is this the first pregnancy?	No	Yes	
Has breastfeeding knowledge or goals been discussed?	Yes	No	
Have other lactation screening tools been used? (Breastfeeding Competency Scale (BCS), other)			
Is there previous breastfeeding experience?	Yes	No	
Is there a history of breastfeeding difficulties?	No		**Yes**
If yes, what was the issue and how was it resolved?			

Breast and Nipple Questions

Note: A care plan should be in place prior to birth if there are any breast/chest or nipple complications or concerns**.**

Have there been noticeable breast changes during this pregnancy?	Yes		**No**
Is there diagnosed breast hypoplasia? Left Right Both	No		**Yes**
Is there any documented trauma, burns, or radiation to breasts?	No		**Yes**
Is there a history of breast or chest surgery?	No		**Yes**
If yes, date and type:<br>(mastectomy, breast augmentation, breast reduction with or without nipple removal, other)			
If yes, place of incision(s) (axillary, subareolar, inframammary, other):			
Is there normal nipple sensation/reaction since surgery?	Yes		**No**
Inverted nipples that do not evert with compression or have graspability?	No		**Yes**

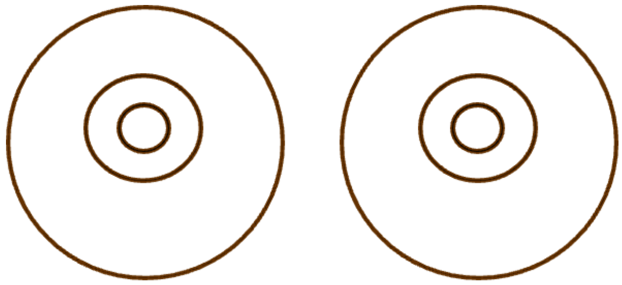

R L

☐ **Refer for Lactation Consultant Care**
☐ **Refer for Breastfeeding Education**
